# Effect of sodium silicate on Portland cement/calcium aluminate cement/gypsum rich-water system: strength and microstructure

**DOI:** 10.1039/c8ra09901d

**Published:** 2019-03-29

**Authors:** Zhiming Wang, Yuning Sun, Shuo Zhang, Yonglong Wang

**Affiliations:** School of Energy Science and Engineering, Henan Polytechnic University Jiaozuo 454002 China sunyn639@126.com; State and Local Joint Engineering Laboratory for Gas Drainage and Ground Control of Deep Mines, Henan Polytechnic University Jiaozuo 454000 China

## Abstract

In this investigation, sodium silicate (SS) was mixed into rich-water (RW) materials consisting of Portland cement, calcium aluminate cement and gypsum for improved mechanical properties. The RW materials containing different amounts of SS were characterized by the compression test, mercury intrusion porosity, scanning electron microscopy, X-ray diffraction and Fourier transform infrared spectroscopy. The results demonstrated that with the increase of SS additions, the early strength of the RW materials increases, and the long-term strength retrogression of the RW materials can be inhibited when the SS content is above 3%. Pore structures of the RW materials are improved significantly due to the filling effect of the calcium silicate hydration (C–S–H) gel from a reaction between silicate ions and Ca(OH)_2_, thus increasing the early strength of the RW materials. For the RW materials containing SS and cured for 0 to 14 days, there are more hexagonal hydrates including CaO·Al_2_O_3_·10H_2_O (CAH_10_) and 2CaO·Al_2_O_3_·8H_2_O (C_2_AH_8_), more C–S–H gel and less ettringite crystals, which is of benefit to the strength of the material. The strength retrogression can be attributed to phase conversions from hexagonal hydrates (CAH_10_ and C_2_AH_8_) to cubic ones (3CaO·Al_2_O_3_·6H_2_O) with lower intercrystal bonding forces. Furthermore, this phase conversion is inhibited effectively by the chemical reaction of silicate ions and CAH_10_ (or C_2_AH_8_), improving the long-term strength of the RW materials.

## Abstract 2

After the addition of sodium silicate, the strength of the RW material is improved and the strength retrogression is inhibited.

## Graphical abstract

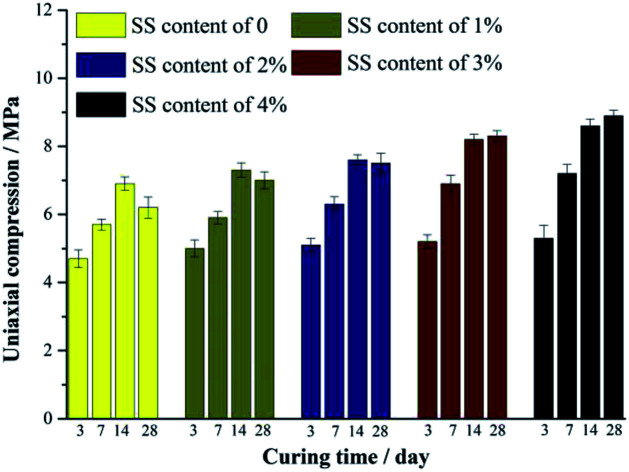

## 1. Introduction

Advanced materials that have properties of high water–solid ratio (W/S), low cost, good strength and durability, are required in the fields of mining engineering, tunnel engineering and oil–gas development. High water materials consisting of sulphoaluminate cement (or its clinker), gypsum and lime have been applied to these engineering fields as filling media.^1,2^ The compressive strength of the high water materials is very low at a high value of W/S, and the strength decreases over time due to water-loss.^3^ To improve the mechanical property of the high water materials, calcium aluminate cement (CAC) was added to sulphoaluminate cement to obtain a novel high water material composed of sulphoaluminate cement, CAC, gypsum, lime and other additives.^4^ However, when stirring water, sulphoaluminate cement, gypsum and lime together by a single-liquid grouting method, the setting time of the high water material pastes is excessively short, which cannot meet the requirements of field-scale applications. To avoid the rapid setting, the high water materials should be divided into two parts for stirring, and then the pastes after stirring are grouted *via* a common pipe by the double-liquid grouting method.^3,4^ This needs complex operations particularly in a small underground space. Therefore, it is meaningful to develop an advanced material that consumes much water and whose slurry can be grouted by a single-liquid grouting method.

Portland cement (PC) has been produced widely in the world, with low cost and rich resources. However, straight PC is not an ideal material to prepare the grouting slurry due to its long setting time, low early strength and obvious shrinkage at a high value of W/S.^5^ For CAC, high early strength is one of the major advantages. Besides, the CAC has other advantages of short setting time, chemical aggression resistance and high temperature resistance.^6^ However, because of the crystalline conversion from the metastable hexagonal CaO·Al_2_O_3_·10H_2_O (CAH_10_) or 2CaO·Al_2_O_3_·8H_2_O (C_2_AH_8_) to cubic 3CaO·Al_2_O_3_·6H_2_O (C_3_AH_6_), the porosity of CAC paste increases and the long-term strength decreases markedly as a consequence.^7^ Meanwhile CAH_10_ or C_2_AH_8_ can react with calcium silicate hydrate (C–S–H) gel to produce 2CaO·Al_2_O_3_·SiO_3_·8H_2_O (C_2_ASH_8_), which is dependent on the availability of C–S–H gel during this reaction process.^6–8^ To obtain advanced materials with short setting time, good strength and low cost, PC and CAC are usually mixed to form PC–CAC binary system.^9–12^ At W/S < 0.6 : 1, when the mass proportion of PC is 5%, the early strength of PC–CAC binary system is improved, and no long-term strength reduction is detected; and when the proportion of PC is 20%, the long-term strength decreases significantly.^9^ And, when the mass ratio of CAC is 20%, the early strength of PC–CAC binary composite material is higher than that of pure PC, and the strength rises over time.^11^ In addition, it was reported that there is an enhancement on the strength of materials consisting of PC or CAC after the addition of sodium silicate (SS).^13–15^

The studies mentioned above provide some important guidance to the novel PC–CAC binary composite materials at low values of W/S. However, there were few studies conducted under rich-water conditions (W/S > 1 : 1). Our prior experimental results have shown that the long-term strength of the rich-water (RW) material composited of PC, CAC and gypsum at relatively high W/S values decreases over time.^16^ In the present study, SS was mixed to improve the mechanical properties of the RW material at the W/S value of 1.2 : 1, and the effects of SS on the RW material were investigated. We tested the compressive strength of RW materials containing different amounts of SS. Whilst, the mercury intrusion porosity (MIP), scanning electron microscopy (SEM), X-ray diffraction (XRD) and Fourier transform infrared spectroscopy (FT-IR) were employed to characterize the microstructures of RW materials. Finally, the strength evolutionary mechanism of the RW material was discussed, which could contribute to the future study on this sort of material.

## 2. Experimental procedures

### 2.1. Materials and sample preparation

**Fig. 1:**
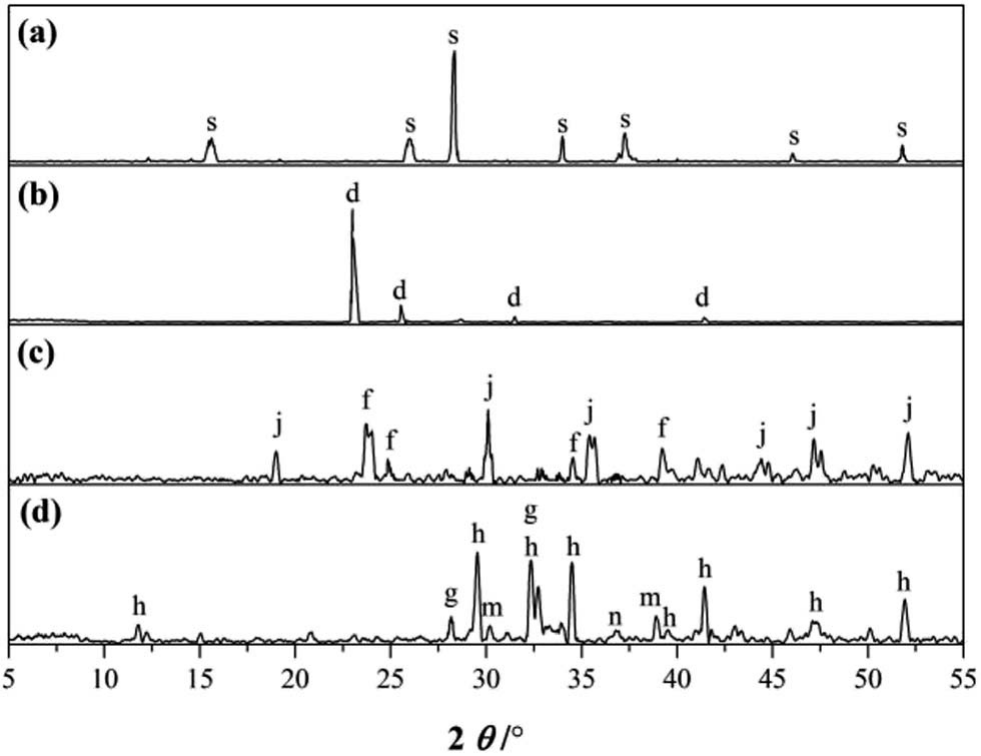
XRD patterns of the starting materials: (a) SS, (b) gypsum, (c) CAC and (d) PC. (s: sodium silicate, d: CaSO_4_·2H_2_O, j: CaO·A_2_O_3_, f: CaO·2A_2_O_3_, h: 3CaO·SiO_2_, g: 2CaO·SiO_2_, m: 3CaO·A_2_O_3_, n: CaO).

Starting materials used in this investigation include PC, CAC, gypsum and SS. PC is produced in Qian-ye Co. Ltd, China, and CAC is supplied by Hua-yan Co. Ltd, Jiaozuo, China. Gypsum with purity of 95% and SS (Na_2_SiO_3_·9H_2_O) with Na_2_O content of 28.0–30.0% are analytical reagents from Kermel chemical agent Co. Ltd, Tianjin, China. Chemical compositions of PC, CAC and gypsum are listed in Table 1. And, Fig. 1 shows the XRD patterns of the starting materials.

**Table 1:** Chemical compositions of PC, CAC and gypsum

Material	Oxides (wt/%)							
	CaO	SiO_2_	Al_2_O_3_	SO_3_	Fe_2_O_3_	K_2_O	MgO	Na_2_O
PC	61.67	21.75	5.57	2.36	3.58	0.87	2.21	0.43
CAC	37.4	0.25	61.4	0.06	0.30	—	—	0.27
Gypsum	26.73	18.56	6.37	28.52	2.37	0.51	1.42	—

The experimental program is based on the study of four designs of the hardened RW paste. Mass proportions of PC, CAC and gypsum in RW are fixed at 65.5%, 14.5% and 10%, respectively. SS, as an additive blended in the RW material, accounts for 0, 1%, 2%, 3% and 4% of the total mass of the RW material, respectively.

According to GB/T 1346-2011, at W/S of 1.2 : 1, the paste was stirred twice in a slurry agitator.^17^ The paste was casted into hollow cylinder moulds (radius of 50 mm and height of 100 mm), and demoulded after 24 hours to prepare cylindrical samples. All the cylindrical samples were cured at (20 ± 2) °C and humidity of (60 ± 5)% until they reached the test ages (3 day, 7 day, 14 day and 28 day).

### 2.2. Measurements

#### 2.2.1. Compressive strength

Adopting RMT-150 mechanical experimental test system (U.S.), the cylindrical samples cured for 3 days, 7 days, 14 days and 28 days were tested, respectively. Loading was set on the sample axially, and there was no confine radially. At a rate of 0.1 kN s^−1^, compressive strength test was conducted until the sample cracked. The compressive strength value was taken to be the means found for three same samples.

#### 2.2.2. MIP measurement

MIP is used to measure the volume of mercury driven into pores of materials at applied pressure. Surface tension of mercury is high and contact angle between mercury and solid is larger than 90°, thus the mercury is a non-wetting substance that only enters pores at the applied pressure.^18^ For materials with low elastic modulus, its volumes of pores can be measured accurately by MIP.^19,20^ In this study, MIP was employed to study the pores in the RW materials. The relation between the applied pressure *p* (MPa) and the pore diameter *d* (m) can be expressed *via* Washburn equation (eqn (1)).^21^

1 *pd* = −4*σ* cos *θ*

where *σ* is the surface tension of mercury (N m^−1^) and *θ* is the contact angle between mercury and solid. Generally, the value of term 4*σ* cos *θ* in eqn (1) is assumed as 1500 MPa nm. By eqn (1), we can obtain the pore distribution of the RW materials.

MIP tests were conducted in an AutoPore IV 9505 porosimetry (U.S.), whose applied pressure ranges from 0 to 228 MPa and measurable diameter of pore is 5 nm to 1000 μm. Cubic specimens (2.5 × 2.5 × 2.5 cm) sliced from the undamaged cylindrical samples that cured for 3 days, 14 days and 28 days, were prepared. Then, the cubic specimens were dried in a vacuum-oven at a low temperature of 30 °C. This temperature selected was to avoid damaging the microstructure of the samples.

#### 2.2.3. SEM

Unharmed cubic bulks (0.5 × 0.5 × 0.5 cm) were cut from the cylindrical samples that cured for 3 days and 28 days, respectively. Then, the cubic bulks were soaked in absolute ethyl alcohol for 48 hours to terminate hydration. Finally, the cubic bulks were dried in a vacuum-oven at 30 °C until there is no change in weight. To improve the surface conductivity, the cubic bulks were treated with spray-gold in the ion sputtering instrument (GVC-1000). Then, SEM (JSM-6390/LV, Japan) was used to study the micromorphology of the RW material under a high vacuum condition. Due to the well-conductivity of the surface after the treatment of spray-gold, we chose 15 kV as the acceleration voltage. The secondary electron imaging (SEI) mode was adopted to obtain the micromorphology of the RW material. And, at a magnification of 4000, clear SEM images could be obtained.

#### 2.2.4. XRD and FT-IR measurements

The cylindrical samples cured for 3 days, 14 days, and 28 days were broken into bulks. These bulks soaked in absolute ethyl alcohol for 48 hours to stop the material from hydration. After that, the bulks were dried in a vacuum oven until there was no change in weight to ensure the elimination of ethyl alcohol. Then, the bulks dried were grounded to a very fine powder in a ball grinding mill.

Adopting X-ray diffractometer (D8 Advance, Bruker, Germany) with a 35 kV, 50 mA copper anode X-ray tube and a Cu Kα radiation, the fine powder was examined to determine the hydration products in different ages. Scanning was conducted between 5° and 55° with a 2*θ* increment of 0.02° s^−1^.

The fine powder (2 mg) for FT-IR analysis was mixed with dry KaBr powder (200 mg), and driven into disks. The infrared spectra of the disked powder were recorded by VERTEX 70 Fourier transform infrared spectrometer (Bruker, Germany) in the region from 4000 cm^−1^ to 400 cm^−1^ at a resolution of 4 cm^−1^. And the powder was scanned 32 times. To eliminate the impact of vaporous water and carbon dioxide in the spectrometer, we collected the spectrum before putting the sample in the sample cell, which is background spectrum indicating the infrared absorption of the vaporous water and carbon dioxide in the spectrometer. By subtracting the background spectrum, the real spectra of the samples could be obtained. Further, the variations of bonds can be analyzed by peak areas of FT-IR.^22–24^ In this paper, the peak areas were calculated by peak-differentiating-fitting method combined with Gauss equation. The peak-differentiating-fitting method includes a process of baseline finding-creating, baseline subtracting, peak finding, peak fitting and peak area calculating. What's more, it is required that the correlation coefficients of fitting and experimental curves should be above 0.90.

## 3. Results

### 3.1. Evolution of strength

**Fig. 2:**
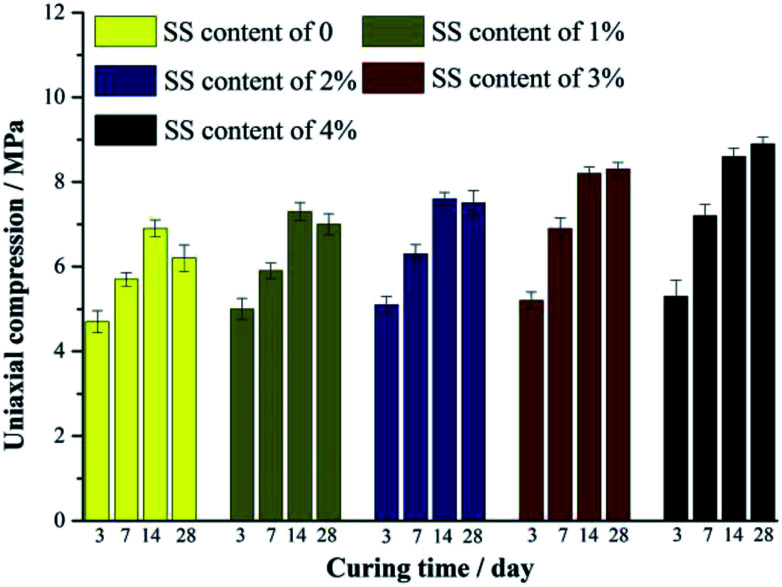
Compressive strength of RW materials with SS additions of 0, 1%, 2%, 3% and 4%. All the samples are cured for 3 day, 7 day, 14 day and 28 day, respectively.

Fig. 2 shows the compressive strength of the RW materials containing different amounts of SS. When the addition of SS is 0, the compression of the RW material on 3 day is 4.71 MPa. The RW materials containing SS exhibit a relatively high compressive strength on 3 day, and for SS contents of 1%, 2%, 3% and 4%, the corresponding strengths are 5.08 MPa, 5.11 MPa, 5.19 MPa and 5.26 MPa, respectively. For all the samples, the compressive strength increases until 14 day, and the strength of the RW materials containing SS is likewise higher than that of the RW without SS. It indicates that the early strength of the RW materials is improved by blending SS.

For the RW materials with SS contents of 0, 1% and 2%, their compressive strength decreases during 14 day to 28 day. However, this kind of strength retrogression reduces with the increase in the content of SS. Until the content of SS increases to 3% at least, compressive strength retrogression does not appear during 14 day to 28 day, indicating that the long-term strength retrogression of the RW material can be inhibited effectively by raising the mixing amount of SS.

### 3.2. Pore structure and micromorphology

**Fig. 3:**
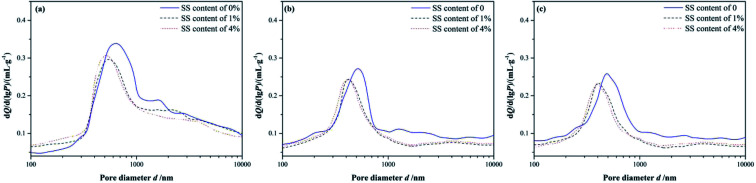
Differential pore size distributions of RW materials with SS mixing amounts of 0, 1% and 4%. (a) Curing time of 3 day. (b) Curing time of 14 day and (c) curing time of 28 day.

The differential pore size distributions of the RW materials with different mixing amounts of SS are shown in Fig. 3. The pore diameters of the RW materials range from 100 nm to 10 μm. And, all the samples have differential peaks at pore diameters in the range of 400–630 nm. The pore sizes corresponding to the peaks reduce over time, the number of large pores (*d* > 1000 nm) gradually decreases, and the number of small pores (*d* < 300 nm) increases instead. These illustrate that the structure of the RW material became more compact from 3 day to 28 day, which is not consistent with the pure CAC paste porosity variation over time reported before.^7,25–27^

On 3 day of hydration and for the hardened RW paste containing no SS, the most probable pore diameter that corresponding to the peak is about 619 nm. And, for the RW materials containing SS of 1% and 4%, the most probable pore diameters are about 552 nm and 516 nm, respectively. It indicates that the pore structure of the RW materials can be improved significantly by mixing SS at early hydration stage. After 14 day's hydration, the most probable pore diameters are 530 nm, 425 nm and 414 nm corresponding to the RW materials containing SS of 0, 1% and 4%, respectively. When curing period of 28 days, the most probable pore diameters of the RW materials are 486 nm, 408 nm and 401 nm corresponding to SS content of 0, 1% and 4%, respectively. Therefore, the difference in the most probable pore sizes of the RW materials between blending with SS and mixing no SS is notable. However, the most probable pore sizes are close for the RW materials containing SS of 1% and 4%. What's more, the difference in the most probable pore size of the RW materials between blending 1% SS and 4% SS reduces over time.

**Fig. 4:**
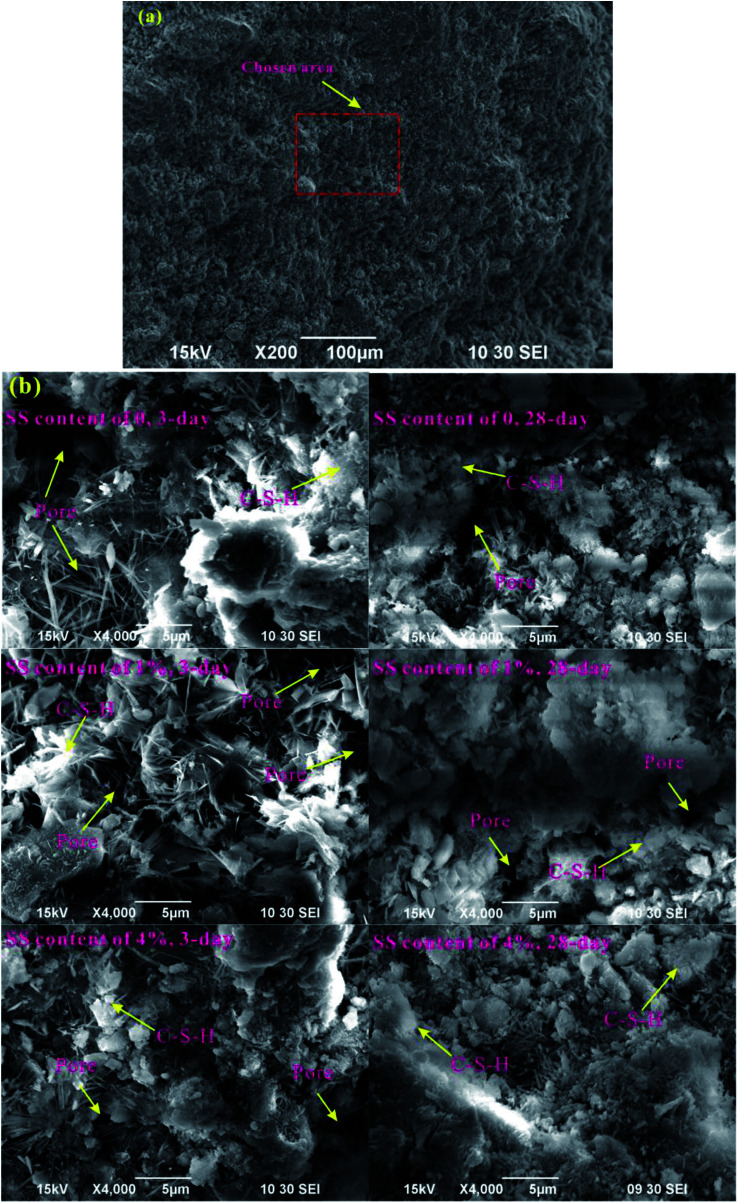
Micromorphology of RW materials. (a) Overview SEM image at a magnification of 200. (b) SEM images at magnification of 4000 showing the micromorphology of the RW materials with SS additions of 0, 1% and 4%, and the RW materials are cured for 3 day and 28 day.

For a further illustration of microstructures of the RW materials and a verification of the MIP results, the micromorphology of the RW materials cured for 3 days and 28 days is shown in Fig. 4. An overview-SEM image at a magnification of 200 is plotted in Fig. 4a, indicating that the chosen areas are representative. And, Fig. 4b shows the SEM images at a magnification of 4000. On 3 day, the number of needle-shaped crystals of the RW paste without SS is more than that of the RW material with SS content of 4%; and for the RW pastes containing SS of 1% and 4%, the pores are filled by amorphous C–S–H gel, leading to a lower porosity. On 28 day, the numbers of the needle-shaped crystals both decrease for the RW materials with and without SS, and the pores are filled by the C–S–H further. Besides, the pore size of the RW material without SS is larger than that of the RW material containing SS of 4%, which is in agreement with the experimental results of MIP.

### 3.3. Mineral compositions and distribution

**Fig. 5:**
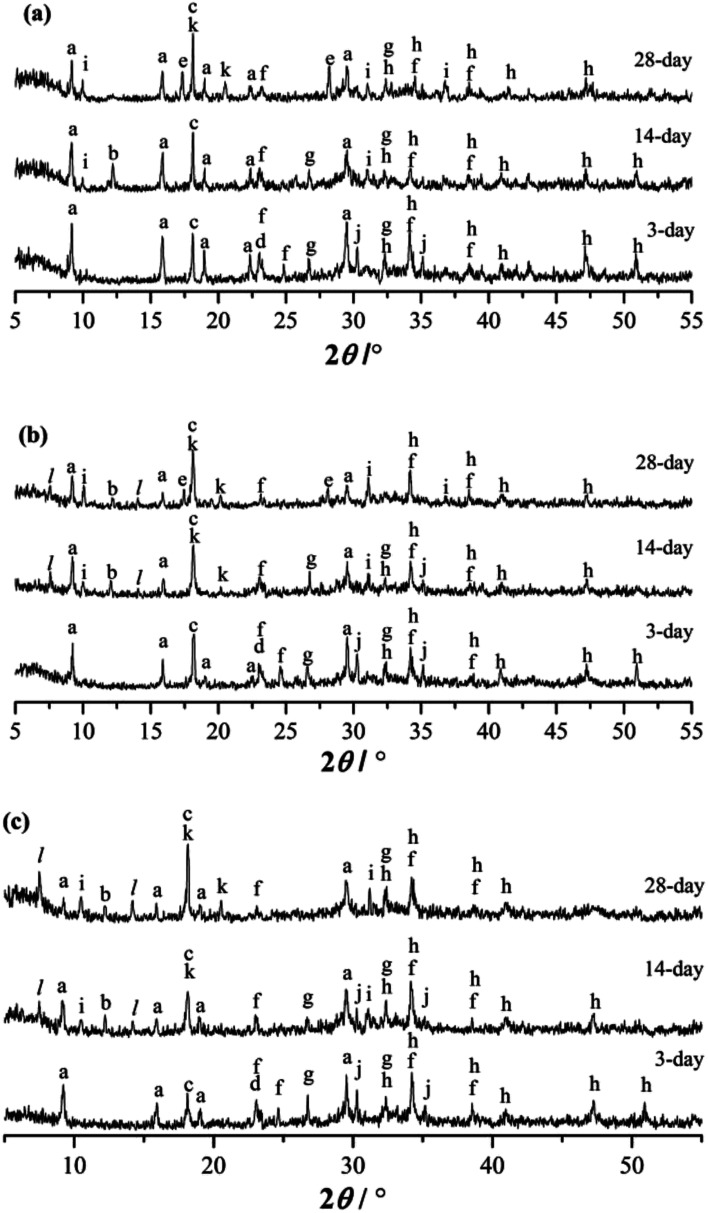
XRD patterns of RW materials hydrated for 3 day, 14 day and 28 day. (a) SS addition of 0. (b) SS addition of 1%. (c) SS addition of 4%. (a: AFt, b: CAH_10_, c: CH, d: CaSO_4_·2H_2_O, e: C_3_AH_6_, f: CA_2_, g: C_2_S, h: C_3_S, i: AFm, j: CA, k: AH_3_, l: C_2_ASH_8_) When SS content is 1%, the categories of the hydration products on 3 day are the same with those of the RW materials containing no SS. However, the diffraction signal of AFt is much lower, illustrating that the hydration process of CA_*x*_ is delayed by mixing SS, and this finding agrees with Ding (1995, 1996).^32,34^ When hydrated for 14 day, the diffraction intensity of CH is lower than the material without SS; and there appear diffraction peaks at *d* = 11.75 Å, 6.24 Å, reflecting the formation of C_2_ASH_8_. According to the studies carried out by Y. Jeong (2018), L. Xu (2017), M. Okoronkwo (2016) and T. Matschei (2007), C_2_ASH_8_ can be also detected at the similar peak positions in the calcium sulfoaluminate–belite cement–gypsum, calcium aluminate cement–Portland cement–anhydrite, Portland–CASH or CASH systems.^10,35–37^. On 28 day, there appears the diffraction signal of C_3_AH_6_, but its diffraction intensity is weaker than that in the RW material without SS.

The XRD patterns of the RW materials containing different amounts of SS are shown in Fig. 5. By phase determinations and by reference to the XRD patterns showing the un-hydrated phases in the starting materials (Fig. 1), we can find that when SS content is 0, in the XRD patterns of the RW materials that hydrated for 3 day, there are significant diffraction signals of gypsum (CaSO_4_·H_2_O), tricalcium silicate (C_3_S), dicalcium silicate (C_2_S), monocalcium aluminate (CA) and calcium dialuminate (CA_2_), reflecting the incomplete hydration of the minerals of PC–CAC–gypsum ternary system. However, the tricalcium aluminate (C_3_A) can't be detected, indicating a complete hydration of C_3_A before 3 day. Besides, on 3 day, significant diffraction signals of ettringite (AFt) crystals can be detected at *d* = 9.63 Å, 5.57 Å, 3.97 Å, 3.02 Å, and the calcium hydroxide (CH) can be found at *d* = 5.57 Å. We don't find any clear diffraction signals of C–S–H gel, due to its amorphous property.^28,29^ However, amorphous C–S–H gel can be found in Fig. 4b. After hydration for 14 days, the height of AFt diffraction peak reduces, and calcium monosulphoaluminate (AFm) can be detected at *d* = 8.83 Å, 2.87 Å, this is because of the inadequate sulfate ions in the ternary paste.^30^ At *d* = 7.92 Å, there appears a diffraction signal of CAH_10_; however, no diffraction signal of C_2_AH_8_ can be detected, which could be caused by the weak diffraction peak of C_2_AH_8_ or the overlapping diffraction peaks of C_2_AH_8_ and AFt.^31,32^ According to C. Evju (2001), when there are adequate sulfate ions, the calcium aluminate-type minerals (CA_*x*_) will react with sulfate ions; and the reaction between CA_*x*_ and water will not begin until the amount of sulfate ions reduces to a low level. Therefore, the formation of AFt is much earlier than CAH_10_ and C_2_AH_8_.^33^ Besides, on 14 day, the diffraction peak of CH is much higher than that on 3 day, indicating the continuous hydration of calcium silicate-type minerals (CS_*x*_), then it can be inferred that there will produce new C–S–H (gel) between 3 day to 14 day. The diffraction peak of CAH_10_ disappears on 28 day, instead the diffraction signals of C_3_AH_6_ and gibbsite (AH_3_) appear at *d* = 5.16, 3.18 Å and *d* = 5.55 Å, 4.36 Å, respectively.

When SS content is 4%, compared with the RW materials containing SS of 0 and 1%, the diffraction signal of AFt is the lowest at the same hydrated stages. On 14 day and 28 day, there is no obvious diffraction peak of C_3_AH_6_. When hydrated for 28 days, the diffraction intensity of C_2_ASH_8_ in the RW material with SS content of 4% is much higher than that of the RW material with SS mixing amount of 1%, revealing much more formation of C_2_ASH_8_ in the RW with SS content of 4%.

**Fig. 6:**
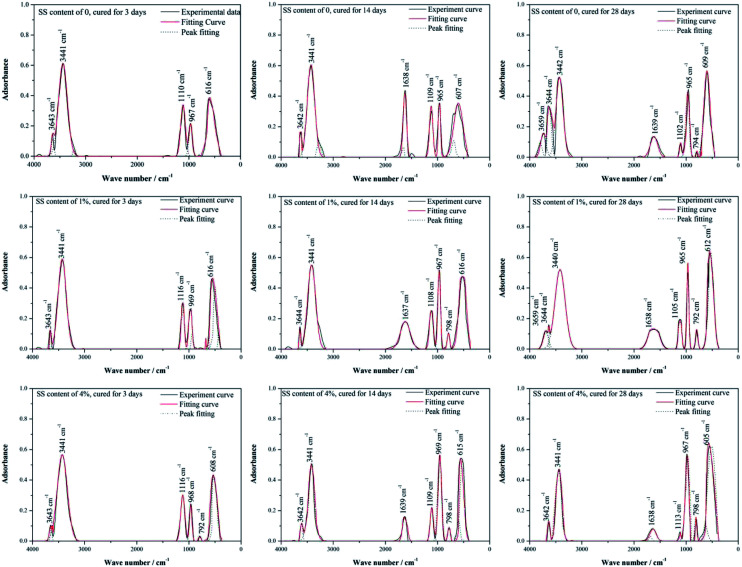
Peak fitting of FT-IR spectra of RW materials containing SS amounts of 0, 1% and 4%. The curing time of the materials is 3 day, 14 day and 28 day.

Further, FT-IR was employed to measure the adsorption bands at characteristic wavelength of chemical bonds that vibrate independently, to determine the chemical compositions of materials and to supplement the XRD results. In Fig. 6, the IR spectra for the RW materials containing different amounts of SS can be divided into a high wave number region (4000–1200 cm^−1^) and a low wave number region (1200–400 cm^−1^). In high wave number region, it is detected an O–H stretching vibration at around 3659 cm^−1^ (W_1_), indicating the formation of C_3_AH_6_;^31^ at 3642–3644 cm^−1^ (W_2_), there appear the O–H vibration bonds induced by CH.^38^ An adsorptive-water adsorption region can be found at 3441 cm^−1^ (W_3_), due to the existence of AFt;^38,39^ and bending vibration bands that induced by the H_2_O or OH of CAH_10_ appear at 1637–1639 cm^−1^ (W_4_).^31,40^ In low wave number region, the adsorption bands at 1102–1116 cm^−1^ (W_5_) is caused by the asymmetric vibration of SO_4_^2−^ existed in AFt, and the bending vibrations at around 605–616 cm^−1^ (W_8_) are also induced by the bands in SO_4_^2−^;^41^ at wave number ranging from 965 cm^−1^ to 969 cm^−1^ (W_6_), there appear stretching vibrations induced by silicate/aluminate-oxide structures;^31^ and the adsorption bonds around 792–798 cm^−1^ (W_7_) are attributed to the symmetric stretching vibration of Si–O–Si.^42^

Table 2 lists the area percentages of fitted peaks calculated using Origin 8.0, which can reflect the content of minerals to some extent. For the RW materials without SS, the peak area percentages at W_2_, W_3_, W_5_, W_6_, W_8_ are 2.92%, 45.26%, 17.99%, 9.44%, and 24.39% on 3 day, respectively. From 3 day to 14 day, there shows an increase trend in the area percentages at W_2_, W_6_ and W_8_, but it decreases in the area percentages at W_3_ and W_5_. It indicates the increase of the amount of the O–H bonds in CH and Si/Al–O bonds in C–S–H and the decrease of the amount of AFt, which confirmed the XRD findings. The peak area percentages on 3 day and 14 day are both 0 at W_1_, indicating no formation of C_3_AH_6_; the peak area percentages at W_4_ are 0 and 12.49% on 3 day and 14 day. However, on 28 day, the peak area percentage at W_1_ raises to 2.87% and the peak area percentage at W_4_ reduces to 5.74%, which illustrates the formation of C_3_AH_6_ and the decrease of CAH_10_.

**Table 2:** Adsorption peak area-percentage by curve fitting

Samples	Curing time	Area percentage/%							
		W_1_	W_2_	W_3_	W_4_	W_5_	W_6_	W_7_	W_8_
RW	3 day	0	2.92	45.26	0	17.99	9.44	0	24.39
	14 day	0	3.07	35.83	12.49	10.36	10.73	0	27.52
	28 day	2.87	7.71	28.05	5.74	2.02	17.77	1.06	34.78
RW + 1% SS	3 day	0	2.04	41.94	0	13.98	13.36	0	28.68
	14 day	0	2.98	31.32	9.94	9.23	17.27	1.51	31.75
	28 day	2.06	3.54	25.38	6.03	3.97	20.58	2.76	35.68
RW + 1% SS	3 day	0	1.07	38.63	0	13.96	13.16	0.99	32.19
	14 day	0	1.21	28.05	5.31	6.88	22.98	1.86	33.71
	28 day	0	2.04	23.74	2.95	0.76	27.14	2.85	40.52

When SS contents of 1% and 4%, compared with the RW materials without SS, the peak area percentage at W_2_ decreases and that at W_6_ increases on 3 day, respectively, showing much more formation of C–S–H, due to the reaction (eqn (2)).^43^ It can verify the porosity lowing induced by the filling effect of C–S–H gel.

2 CH + SiO_3_^2−^ → C–S–H

For the RW material with SS content of 1%, the area percentage at W_1_ is 2.06% on 28 day, which is lower than that in the RW material without SS. However, when the SS content in the RW material increases to 4%, the area percentages at W_1_ are all 0 on 3 day, 14 day and 28 day, indicating no formation of C_3_AH_6_. Besides, for the RW materials with SS additions of 0, 1% and 4%, the area percentage at W_4_ decreased from 14 day to 28 day, indicating the consumption of CAH_10_. These are all consistent well with the XRD results (Fig. 5).

## 4. Discussion

### 4.1. Impact of porosity on strength

**Fig. 7:**
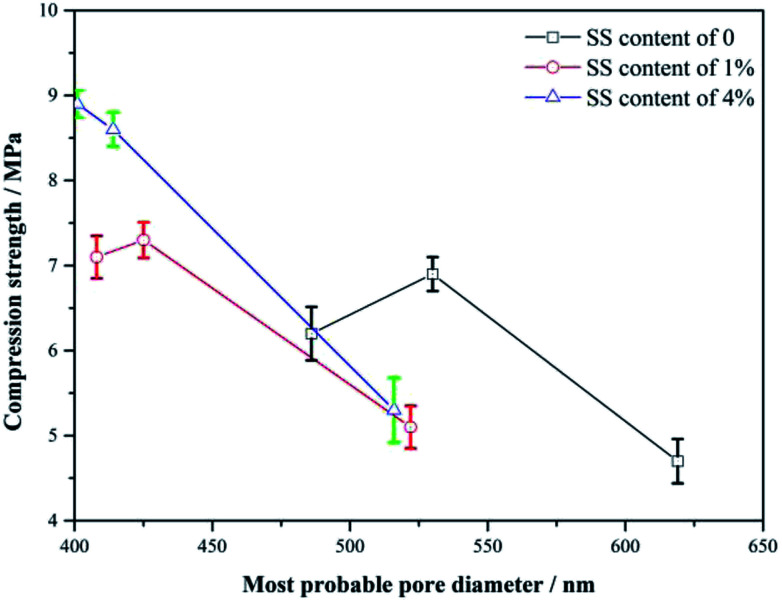
Variation of strength with diameter of the most probable pore in the RW materials. From right to left, the three points (the compressive strength – most probable diameter) of each curve correspond to the RW materials hydrated for 3 day, 14 day, and 28 day.

Fig. 7 shows the variations of the strength with the diameter of the most probable pores for the RW materials with SS additions of 0, 1% and 4%. From 3 day to 14 day, the less the most probable pore diameter, the higher the strength of the RW materials with or without SS, indicating the strength enhancements may be caused by the decrease in the pore size in the materials, which agrees well with Matusinović (2003).^25^ However, the continuous decrease in the pore size cannot explain the strength retrogression of the RW materials containing SS of 0 and 1%. Therefore, it needs a further discussion on the strength evolution mechanism by analyzing hydration products.

### 4.2. Impact of phase transformations on strength

Generally, formations of CAH_10_ and C_2_AH_8_ are critical factors influencing the long-term strength of hardened CAC paste.^9,10,31^ However, due to the continuous hydration of CA_*x*_ minerals or the increase in temperature, the hydration products (CAH_10_ and C_2_AH_8_) of CAC would transform to C_3_AH_6_ (eqn (3)); furthermore, under the conditions of high alkalinity, CA_*x*_ can directly combine with water to form C_3_AH_6_ (eqn (4)).^31,32^3
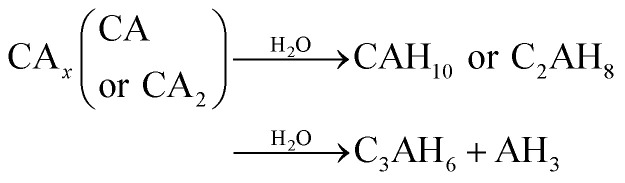
4
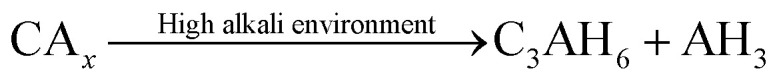


CAH_10_ and C_2_AH_8_ are meta-stable hexagonal hydrates, and C_3_AH_6_ is a stable cubic hydrate.^10,11,31^ And, the intercrystal bonding force of the hexagonal hydrate is larger than that of the cubic one. Therefore, based on the results of XRD and FT-IR, the conversions of CAH_10_ and C_2_AH_8_ to C_3_AH_6_ can explain the strength decline of the RW materials with SS content from 0 to 2%.Besides, at initial hydration phase, when there exists SO_4_^2−^, CA_*x*_ will hydrate and produce AFt crystals first (eqn (5)).^17^ However, excessive formations of AFt at early hydration stage will inhibit the hydration process of calcium silicate type minerals (CS_*x*_); then, the formation of C–S–H (gel) decreases to induce a lower early strength of the RW materials.^44^5
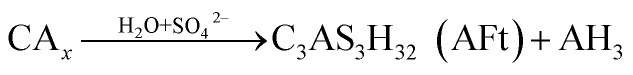


For the RW material without SS hydrated for 14 day, the diffraction intensity of CH is larger than that on 3 day; besides, the peak area become larger at around 967 cm^−1^. Thus, it indicated the continuous reaction between C_*x*_S and water (eqn (6)) during 3 day to 14 day.^45,46^

6 C_*x*_S + H_2_O → C–S–H (gel) + CH

**Fig. 8:**
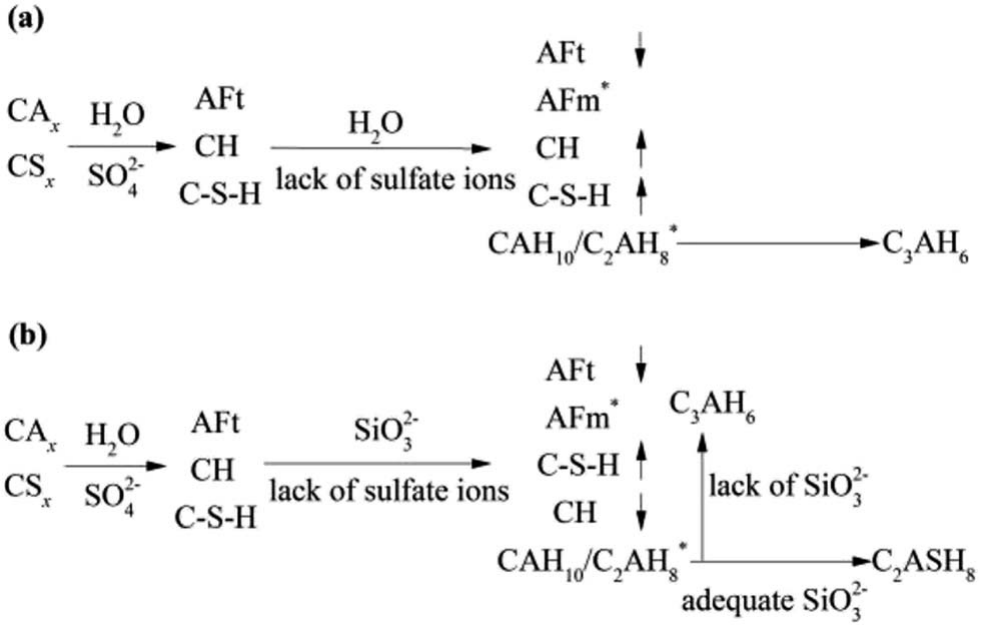
Phase transformation processes in the RW materials (a) without SS and (b) containing SS. Symbols of ↑ and ↓ represent mass increase and decrease, and * represents new hydrates.

During 0 to 14 day, the hydrates, CAH_10_, C_2_AH_8_ and C–S–H (gel) are of benefit to the strength, which can explain the increase in strength of the RW material without SS. However, on 28 day, it can be detected the conversion of CAH_10_ and C_2_AH_8_ to C_3_AH_6_, inducing a lower long-term strength of the RW material without SS for the lower intercrystal bonding force of C_3_AH_6_. The phase variations in the RW material without SS is plotted in Fig. 8a.

The variation of hydrates in the RW materials containing SS is plotted in Fig. 8b. Combining with XRD and FTIR results, for the RW materials with SS, the hydration rate of CA_*x*_ is inhibited to cause a decrease in the formation of AFt;^32,34^ then, the hydration reaction of CS_*x*_ and the reaction between SS and CH are both accelerated to produce much more C–S–H (gel) to improve the early strength of the material.^15^ Due to sulfate ions reducing with further hydration, the CA_*x*_ minerals reacts with water to form CAH_10_ and C_2_AH_8_, this process is similar with that of the RW material without SS. After that, due to the existence of SS, CAH_10_ and C_2_AH_8_ could react with silicate ions to form the stable phase of C_2_ASH_8_ before the conversion from the hexagonal crystal to the cubic one (eqn (7)),^32^ which was also reported by M. Heikal (2017) and J. M. Mercury (2007).^6,17^ Then, the conversion from the hexagonal crystal to the cubic one could be inhibited. C_2_ASH_8_ is a stable hexagonal crystal, which cannot induce the strength retrogression of the RW material. On 28 day, C_3_AH_6_ can be also detected in the RW material with SS mixing amount of 1%, it is because SS is consumed completely, which cannot react with all the hydrates of CAH_10_ and C_2_AH_8_, and the remaining CAH_10_ and C_2_AH_8_ are conversed to C_3_AH_6_. What's more, based on Fig. 5 and 6, the formation amount of C_3_AH_6_ in the RW material with SS of 1% is less than that in the RW material without SS, thus, the decrement of strength is less.7
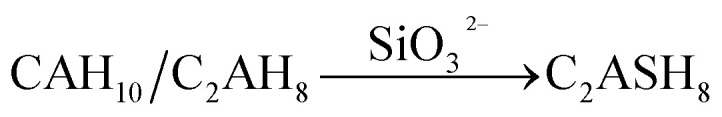


According to the results of XRD and FT-IR, from 3 day to 14 day, the amounts of C–S–H (gel) and C_2_ASH_8_ in the RW materials containing SS of 4% are more than those in the RW material containing SS of 1%, which can explain the higher strength. Besides, there is no evidence of the formation of C_3_AH_6_ from 3 day to 28 day, indicating the silicate ions is so adequate that can consume CAH_10_ and C_2_AH_8_ fully. Therefore, the decrease in the long-term strength of the RW material can be inhibited effectively.

## 5. Conclusions

By way of summary, according to the findings of the strength and micro-structural characterizations of the RW materials containing different amounts of SS, the main relevant conclusions of this study are listed below:

(1) The strength of the RW material could be enhanced by mixing with SS. And the more the SS content, the higher the strength. However, when the addition of SS less than 3%, the long-strength retrogression is existed in the RW material hydrated for 28 days. When SS mixing amount is above 3%, the strength retrogression of the RW material can be inhibited effectively.

(2) The compactness of the RW material is improved by the addition of SS. Compared with the RW material without SS, the RW material containing SS have a lower most probable pore diameter, due to the filling effect of C–S–H (gel) that is produced by the reaction between silicate ions and CH. And, whatever the amount of SS mixed, the compactness of the RW material increases over time, it can probably explain the strength enhancement during 3–14 day, but cannot explain the strength retrogression of the RW materials detected on 28 day.

(3) For the RW materials containing SS and hydrated for 0 to 14 day, there are more hexagonal hydrates and C–S–H (gel) and less AFt crystals, it is of benefit to the strength. However, the phase conversion of the hexagonal hydrates (CAH_10_ and C_2_AH_8_) to the cubic one (C_3_AH_6_) causes the strength retrogression of the RW materials with SS additions of 0 and 1%. Due to the reaction between silicate ions and CAH_10_ and C_2_AH_8_ to form C_2_ASH_8_, the content of C_3_AH_6_ in the RW material containing SS of 1% is less than that in the RW material without SS, that is the reason why the long-term strength decrement of the RW material containing SS of 1% is less than that of the RW material without SS. In addition, for the RW material with SS addition of 4%, the formation of C_3_AH_6_ is inhibited completely, thus, there is no long-term strength retrogression of the RW material.

## Conflicts of interest

There is no conflict to declare.

## Supplementary Material
